# Prevalence of Alpha-1 Antitrypsin Deficiency Alleles in a Lithuanian Cohort of Wheezing Small Children

**DOI:** 10.3390/arm92040028

**Published:** 2024-08-05

**Authors:** Edita Poluzioroviene, Joanna Chorostowska-Wynimko, Sigita Petraitiene, Arunas Strumila, Adriana Rozy, Aneta Zdral, Arunas Valiulis

**Affiliations:** 1 Clinic of Children’s Diseases, Institute of Clinical Medicine, Faculty of Medicine, Vilnius University, Santariskiu Str. 7, LT-08410 Vilnius, Lithuania; sigita.petraitiene@santa.lt (S.P.); arunas.valiulis@mf.vu.lt (A.V.); 2 Department of Genetics and Clinical Immunology, National Institute of Tuberculosis and Lung Diseases, 01-138 Warsaw, Poland; j.chorostowska@igichp.edu.pl (J.C.-W.); adriana.rozy@gmail.com (A.R.); aneta_zdral@interia.eu (A.Z.); 3 Vilnius University Hospital Santaros Klinikos, LT-08406 Vilnius, Lithuania; arunas.strumila@santa.lt; 4 Department of Public Health, Institute of Health Sciences, Faculty of Medicine, Vilnius University, LT-08410 Vilnius, Lithuania

**Keywords:** alpha-1 antitrypsin deficiency, children, wheeze, SERPINA1, COPD

## Abstract

**Highlights:**

**What are the main findings?**

- Alpha-1 antitrypsin phenotypes are linked to both chronic obstructive pulmonary disease in adults and wheezing syndrome in preschool children.
- The frequency of Z* and S* alleles in a cohort of wheezing preschool children is higher than in the general population and similar to that in adults with chronic obstructive pulmonary disease.

**What is the implication of the main finding?**

- Our data open the way for further research on the role of both homozygous and heterozygous variants of alpha-1 antitrypsin in the development of preschool wheezing.
- Stratification of the heterogeneous syndrome of preschool wheezing allows us to search for chronic obstructive pulmonary disease-related wheezing phenotype/endotype and start the disease-specific prophylactic in early childhood.

**Abstract:**

Severe inherited alpha-1 antitrypsin deficiency (AATD) is an autosomal genetic condition linked to chronic obstructive pulmonary disease (COPD). The significance of heterozygous, milder deficiency variants (PiSZ, PiMZ, PiMS) is less clear. We studied AATD genotypes in 145 children (up to 72 months old) with assessed wheezing severity using the Pediatric Respiratory Assessment Measure (BCCH PRAM score). A control group of 74 children without airway obstruction was included. AAT concentration and Pi phenotype were determined from dry blood spot samples using nephelometry and real-time PCR; PiS and PiZ alleles were identified by isoelectrofocusing. Among the wheezers, the Pi*S allele incidence was 2.07% (3 cases) and the Pi*Z allele was 6.9% (10 cases). The Pi*Z allele frequency was higher in wheezers compared to controls (44.8% vs. 20.27%) and the general Lithuanian population (44.8% vs. 13.6%) and was similar to adult COPD patients in Lithuania: Pi*S 10.3% vs. 15.8% and Pi*Z 44.8% vs. 46.1%. No association was found between AAT genotypes and wheezing severity. Finding that wheezer children exhibit a frequency of Z* and S* alleles like that found in adults with COPD suggests a potential genetic predisposition that links early wheezing in children to the development of COPD in adulthood. Larger cohort studies are needed to confirm this finding.

## 1. Introduction

Normal alleles of the *SERPINA1* gene are referred to as PiM, where “Pi” stands for protease inhibitor and “M” denotes medium mobility. The PiM allele produces the standard form and normal levels (1.2–2 g/L) of alpha-1 antitrypsin (AAT) protein, which functions to protect tissues from enzymes released during inflammation, particularly in the lungs [1]. The most common mutant alleles of the *SERPINA1* gene are Pi*Z (Glu342Lys) and Pi*S (Glu264Val). Individuals with two mutated alleles (homozygous or compound heterozygous) have significantly reduced levels of functional AAT and are at much higher risk of various types of liver diseases in both children and adults, as well as lung disease at a young age, and the disorder occasionally occurs as vasculitis, necrotizing panniculitis, or other chronic inflammatory diseases [2]. Nearly 100 percent of the clinical cases of AATD-associated pathologies involve the Pi*Z allele, as PiZZ homozygous or less frequently as compound heterozygous [3]. In clinical practice, pulmonary emphysema, bronchiectasis, and chronic bronchitis are the most frequently associated with PiZZ [4] while the significance of heterozygous variants such as PiSZ, PiMZ, and PiMS is less clear [5,6,7].

Other less common mutant alleles include Pi*F (fast migration) and Pi*I (intermediate migration), which produce AAT with slightly altered function and usually do not cause significant clinical symptoms. Null alleles (e.g., Pi*Q0) result in not measurable or very low levels of AAT and high risk for pulmonary diseases [8]. To date, hundreds of variants of the *SERPINA1* gene have been identified and about 70 of them have been associated with clinical manifestations [1]. 

In this study, we aimed to determine the prevalence of Pi*Z and Pi*S AATD variants in a cohort of Lithuanian small children diagnosed as wheezers of varying severity and compare findings with the control group as well as with data earlier collected from COPD patients in the Central–Eastern European AAT Network and non-disease specific epidemiological studies (SES) performed in our region [9,10]. 

## 2. Materials and Methods

In total, 145 children with an acute wheezing episode were included in the study. In addition, 74 children without respiratory conditions who were hospitalized for various surgical pathology and required a blood sample in preparation for surgery were used as a control group (Table 1). The study was carried out with the permission of the Lithuanian Bioethics Committee (L-16-07/2).

Blood samples were taken on dry blood spot cards and sent to the National Institute of Tuberculosis and Lung Diseases in Warsaw, Poland. Diagnostic tests performed included determination of AAT concentration, phenotyping, genotyping, and DNA sequencing when appropriate. 

Wheezing severity, which was classified into mild, medium, and severe, was calculated using the Pediatric Respiratory Assessment Measure (PRAM). We looked for an association between AAT genotypes and the severity of wheezing.

A common “cold” was diagnosed based on anamnesis and objective examination data. Complaints expressed by parents (fever, runny nose, cough) and objective examination data were evaluated according to the algorithm approved by the hospital, and patients with wheezing syndrome were divided into two groups—children with wheezing when having a cold and children with wheezing without having a cold.

Data for comparison with COPD patients were taken from the CEE A1AT Network, established within the LPP Leonardo da Vinci EU program (2011-1-PL-LEO04-197151) “Introducing standards of the best medical practice for the patients with inherited Alpha-1 Antitrypsin Deficiency in Central Eastern Europe” DBS samples were collected from 328 COPD patients between October 2012 and January 2013. The data were also compared with two previous epidemiological studies conducted with healthy Lithuanian subjects (N = 2491) in determining the general population frequency of PI*S and PI*Z genes [11,12].

## 3. Statistical Analysis

Statistical analysis was performed using IBM SPSS Statistics version 20.0 and Microsoft Excel 365. Continuous and categorical variables were presented as median (interquartile range (IQR) and numbers (percentages %), respectively. Mann–Whitney U or Friedman test was used to compare continuous variables, and χ^2^ test or Fisher’s exact test was used to compare categorical variables. A two-sided *p*-value < 0.05 was considered significant.

Pi*Z and Pi*S frequency is expressed as a frequency per thousand alleles with 95% CI (e.g., 3 Pi*Z alleles were found out of 74 subjects, i.e., 148 alleles, then the frequency per 1000 alleles is calculated as follows: 3 × 1000/148 = 20.27).

## 4. Results

### 4.1. Characteristics of Wheezing Children and Control Group

Children with wheezing episodes were statistically significantly younger compared to children in the control group (Table 1).

The median AAT concentration did not differ between wheezing children and the control group: 144 (IQR 119.5–168) mg/dL in wheezers and 147.5 (IQR 126–165.25) mg/dL in controls, *p* = 0.701. A total of 22.1% of wheezing children had food allergies and 26.9% of wheezing children had atopic dermatitis. The prevalence of food allergies and atopic dermatitis was significantly higher in wheezing children compared to the control group (Table 2). A history of family smoking was more prevalent in the families of wheezing children compared to controls (33.79% vs. 6.76%, *p* < 0.001).

Out of 145 children clinically identified as wheezers, 59 (40.69%) experienced their first wheezing episode, while 86 (59.31%) had recurrent episodes. Children with their first wheezing episode were significantly younger than those with repeated episodes (respectively, 15 (IQR 5–24) months vs. 25 (IQR 12.75–36) months of age, *p* < 0.001). The median AAT concentration did not differ between children with their first wheezing episode and children with repeated wheezing episodes. A significantly higher percentage of children with their first wheezing episode needed hospitalization compared to children with repeated wheezing episodes (91.53% vs. 76.74%, *p* = 0.021), although the length of stay in the hospital did not differ. Other clinical characteristics, such as the severity of wheezing, allergies, family history of smoking, and the presence of concomitant diseases, did not differ significantly between these subgroups (Table 3).

Out of 145 wheezing children, 25 (17.2%) experienced wheezing without having a cold. Children wheezing without a cold were significantly older than those who experienced wheezing only when they had a cold (30 months (IQR 13.5–52) vs. 19 months (IQR 10–30), *p* = 0.009). The median AAT concentration did not differ between children wheezing without having a cold and those wheezing only when they had a cold. A significantly higher percentage of children wheezing when having a cold needed hospitalization compared to children wheezing without a cold (88.33% vs. 56%, *p* < 0.001), but the length of stay in the hospital did not differ. There was no difference in wheezing severity between children wheezing with a cold and those wheezing without a cold. Allergic rhinitis, atopic dermatitis, and a family history of smoking were more frequent among children wheezing without a cold compared to those wheezing only when they had a cold (Table 4).

### 4.2. AAT Genotypes among Wheezing Children and Controls

Among 145 children diagnosed as wheezers, we found a normal PiMM genetic variant of AAT in 129 (88.97%), Pi*S in 3 (2.07%), Pi*Z in 10 (6.90%), and rare mutations were identified in 3 (2.07%) cases. Among the control group’s children, a normal PiMM genetic variant of AAT was found in 68 (91.89%). There was no statistically significant difference in the prevalence of a normal PiMM genetic variant of AAT between children with wheezing and the control group children (*p* = 0.496). The distribution of alpha-1 antitrypsin genotypes in wheezing and control groups is shown in Table 5.

Among the wheezing children, 43 (29.66%) experienced mild wheezing, 65 (44.83%) experienced moderate wheezing, and 37 (25.52%) experienced severe wheezing. No statistically significant difference in AAT concentration and prevalence of AATD alleles was found between groups of patients categorized by disease severity (Table 6).

Plasma concentrations of AAT were in line with AAT genotypes.

The median AAT concentration in PiMM wheezing children (n = 129) was 148.00 (IQR 124.5–170) mg/dL and in the control group (n = 68) was 149.5 (IQR 129.25–167.75) mg/dL.

The median AAT concentration in Pi*S wheezing children (n = 3) was 101 mg/dL and in the control group (n = 3) was 151 mg/dL. 

The median AAT concentration in Pi*Z wheezing children (n = 10) was 95.3 (IQR 91.45–131.5) mg/dL and in the control group (n = 3) was 91.7 mg/dL. The median AAT concentration in wheezing children with rare mutations (n = 3) was 154 mg/dL.

**Figure 1:**
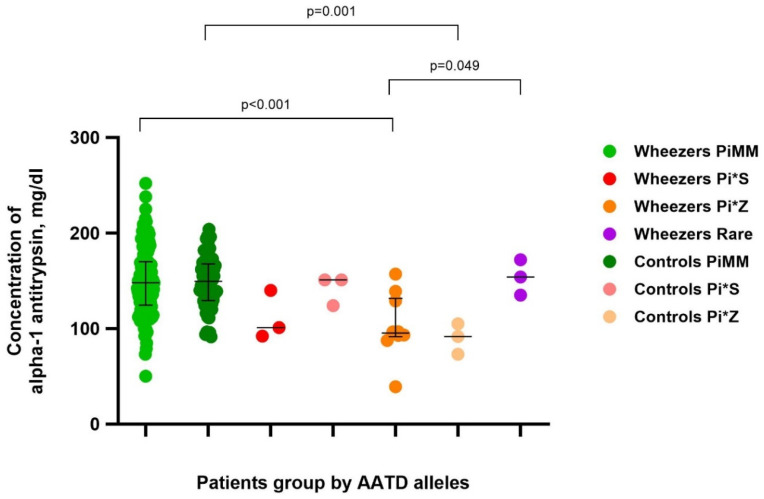
Plasma concentration of AAT in wheezers and controls in groups by genotype.

The levels of AAT in wheezers and controls with deficient variants of AAT are shown in Figure 1.

There was no statistically significant difference in AAT levels between wheezers and controls in groups by genotype.

The data we obtained confirmed the known fact that AAT concentration is lower in cases of deficient genotypes (Figure 1).

### 4.3. Pi*Z and Pi*S Frequencies in Comparison with Other Cohorts

The Pi*Z allele was found to be statistically significantly more frequent in Lithuanian wheezing children than in individuals from Lithuanian non-disease-specific epidemiological studies (NDSES).

The frequency of Pi*S and Pi*Z alleles among wheezing children is close to the frequency of these alleles among COPD patients: Pi*S 10.3 (95% CI: 4.0–16.6) vs. 15.8 (95% CI: 6.92–24.6) and Pi*Z 44.8 (95% CI: 32.1–57.5) vs. 46.1 (95% CI: 31.1–60.9).

The Pi*Z allele in the wheezing group is significantly different from that of the control group (44.8 (95% CI: 32.1–57.5) vs. 20.27 (95% CI: 11.53–29.01). And on the contrary, more Pi*S mutational variants were found in the control group than in the wheezing group (20.27 (95% CI: 11.53–29.01) vs. 10.3 (95% CI: 4.0–16.6) (Table 7).

## 5. Discussion

Childhood wheezing illness appears to be associated with an increased risk of developing COPD in adulthood [13,14,15]. This association may be due to several factors. For example, children with wheezing illnesses may be more sensitive to environmental factors such as tobacco smoke, air pollution, and respiratory infections [16,17,18]. Persistent inflammation from repeated wheezing episodes can lead to structural changes in the lungs, including airway remodeling and reduced elasticity, which are key characteristics of COPD. Overall, the relationship between childhood wheezing and adult COPD highlights the importance of early identification and management of respiratory issues in children to potentially mitigate long-term adverse outcomes on lung health.

Children who experience wheezing may have underlying genetic factors that predispose them to both early-life respiratory issues and the development of COPD later in life. Certain genetic variants, like those in the SERPINA1 gene associated with AATD, can contribute to both childhood wheezing and adult airway disorders. However, the existing data are controversial. For instance, several studies have reported an association between AATD and adult bronchial asthma [19,20,21]. In contrast, the relationship between AATD and childhood bronchial asthma remains debated [22,23,24]. Our study, based on the ALSPAC cohort, did not show an association between SERPINA1 gene polymorphisms and the risk of developing bronchial asthma in school-aged children [25].

Our current results show that the frequency of the Pi*Z allele in wheezing small children is significantly higher than in the control group (44.8% [95% CI: 32.1–57.5] vs. 20.27% [95% CI: 11.53–29.01], respectively). Additionally, the prevalence of the Pi*Z allele in these children (44.8 alleles per 1000) is higher compared to general population estimates based on data from 21 European countries [26,27]. 

Interestingly, we found more Pi*S variants in the control group than in the wheezing group. This observation might be related to the fact that the primary pathologies in the control group, which necessitated surgeries, were conditions such as abdominal wall and inguinal hernias (52.7%), port wine stains (14.9%), benign tumors (8.1%), vascular malformations (8.1%), cystic formations (6.8%), hemorrhoidal nodes (2.7%), and other individual cases (6.8%). It is possible that these pathologies are associated with AATD, as AAT is a major inhibitor of neutrophil proteases and certain metalloproteases involved in remodeling and repairing connective tissue. This connection could explain the higher prevalence of PiS variants in the control group [28,29].

We also compared the frequency of Pi*S and Pi*Z alleles among wheezing children with previously reported data on the frequencies of these alleles in COPD patients from the Central–Eastern European AAT Network and non-disease-specific epidemiological studies performed in Lithuania [9,10]. The frequency of the Pi*S and Pi*Z alleles among wheezing children was like the frequency observed among COPD patients: PiS 10.3% (95% CI: 4.0–16.6) vs. 15.8% (95% CI: 6.92–24.6) and PiZ 44.8% (95% CI: 32.1–57.5) vs. 46.1% (95% CI: 31.1–60.9). Furthermore, the Pi*Z allele was significantly more common in wheezing children compared to data from non-disease-specific epidemiological studies, with a frequency of 44.8% (95% CI: 32.1–57.5) vs. 13.6% (95% CI: 10.7–17.4).

Taken together, findings from our single-center cohort of small children diagnosed as wheezers suggest the necessity of verifying the role of AAT heterozygous mutations in the manifestation of wheezing and the further development of COPD. Understanding how these genetic variations contribute to early respiratory issues can provide valuable insights into the mechanisms underlying both childhood wheezing and adult COPD.

By examining larger and more diverse cohorts, researchers can better determine the specific impact of heterozygous AAT mutations on respiratory health. This includes understanding how these mutations affect AAT levels and lung function from an early age. Moreover, early identification of children with AAT mutations could allow for targeted interventions aimed at preventing the progression of wheezing to more severe respiratory conditions.

## 6. Conclusions

With a clearer understanding of the genetic factors involved, it may be possible to develop preventative strategies or treatments that can mitigate the risk of developing COPD in later life for those identified as genetically predisposed. Overall, ongoing research is crucial to fully elucidate the relationship between AAT heterozygous mutations, early-life wheezing, and the risk of developing COPD, ultimately contributing to better prevention, diagnosis, and treatment strategies.

## Figures and Tables

**Table 1:** Age and gender of wheezing and control children included in the study.

Cases	Median Age (Months, IQR)	Range of Age		Male N (%)	Female N (%)
		Min	Max		
Wheezers	20.00 (11.00–31.50)	2	66	94 (64.83)	51 (35.17)
Controls	53.50 (27.75–69.25)	3	72	43 (58.11)	31 (41.89)

N—number, IQR—interquartile range.

**Table 2:** Clinical characteristics of wheezing children and control group.

Variable	Wheezing Group	Control Group	*p*
AAT concentration (IQR), mg/dL	144 (119.5–168)	147.5 (126–166.25)	0.701
Food allergy, N (%)	32 (22.07)	1 (1.35)	<0.001
Positive ODM or IgE, N (%)	37 (25.52)	3 (4.05)	<0.001
Allergic rhinitis	12 (8.27)	6 (8.11)	0.966
Atopic dermatitis, N (%)	39 (26.89)	2 (2.70)	<0.001
History of family smoking, N (%)	49 (33.79)	5 (6.76)	<0.001

**Table 3:** Clinical characteristics of children with the first and repeated wheezing episode.

Variable	Children with the First Wheezing Episode, N = 59	Children with Repeated Wheezing Episode, N = 86	*p*
Age (IQR), months	15 (5–24)	25 (12.75–36)	<0.001
AAT (IQR), mg/dL	148 (127–164)	139 (114.75–170)	0.431
Need of hospitalization, N (%)	54 (91.53)	66 (76.74)	0.021
Length of stay in hospital, days	3 (3–5)	4 (3–5)	0.957
Wheezing without getting cold, N (%)	2 (3.39)	23 (26.74)	<0.001
Mild wheezing, N (%)	13 (22.03)	30 (34.88)	0.215
Moderate wheezing, N (%)	28 (47.46)	37 (43.02)	
Severe wheezing, N (%)	18 (30.51)	19 (22.09)	
Food allergy, N (%)	11 (18.64)	21 (24.42)	0.410
Positive ODM/IgE, N (%) *	11 (68.75)	26 (52.00)	0.240
Allergic rhinitis, N (%)	3 (5.08)	9 (10.47)	0.361
Atopic dermatitis, N (%)	14 (23.73)	25 (29.07)	0.476
History of family smoking, N (%)	22 (37.29)	27 (31.40)	0.461
Concomitant diseases, N (%)	33 (55.93)	49 (56.98)	0.901

* N = 66 (others did not have an allergy testing).

**Table 4:** Clinical characteristics of children wheezing without having a cold and children wheezing when having a cold.

Variable	Children with Wheezing without Having a Cold, N = 25	Children with Wheezing When Having a Cold, N = 120	*p*
Age (IQR), months	30 (13.5–52)	19 (10–30)	0.009
AAT (IQR), mg/dL	132 (105.5–151.5)	148 (123.25–169)	0.054
Need of hospitalization, N (%)	14 (56.00)	106 (88.33)	<0.001
Length of stay in hospital, days	4.5 (3–7.25)	3 (3–5)	0.063
Mild wheezing, N (%)	8 (32.00)	35 (29.17)	0.785
Moderate wheezing, N (%)	12 (48.00)	53 (44.17)	
Severe wheezing, N (%)	5 (20.00)	32 (26.67)	
Food allergy, N (%)	6 (24.00)	26 (21.67)	0.798
Positive ODM/IgE, N (%) *	7 (28.00)	30 (25.00)	0.054
Allergic rhinitis, N (%)	7 (28.00)	5 (4.17)	0.001
Atopic dermatitis, N (%)	11 (44.00)	28 (23.33)	0.034
History of family smoking, N (%)	14 (56.00)	35 (29.17)	0.010
Concomitant diseases, N (%)	12 (48.00)	70 (58.33)	0.343

* N = 66 (others did not have an allergy testing).

**Table 5:** Distribution of alpha-1 antitrypsin genotypes in wheezing and control groups.

	Total N	PiMM N (%)	Pi*Z N (%)	Pi*S N (%)	Rare N (%)
Wheezing group	145	129 (88.97)	10 (6.90)	3 (2.07)	3 (2.07)
Control group	74	68 (91.89)	3 (4.05)	3 (4.05)	0

**Table 6:** Distribution of AAT alleles between wheezers by disease severity.

Variable	Mild Wheezing	Moderate Wheezing	Severe Wheezing	*p*
AAT concentration (IQR), mg/dL	136 (100–167)	151 (126–185.5)	140 (121–158)	0.129
PiMM, N (%)	38 (29.46)	58 (44.96)	33 (25.58)	n.s.
Pi*S, N (%)	1 (33.33)	1 (33.33)	1 (33.33)	
Pi*Z, N (%)	3 (30.00)	4 (40.00)	3 (30.00)	
Rare, N (%)	1 (33.33)	2 (66.67)	0	
Total N (%)	43 (29.66)	65 (44.83)	37 (25.52)	-

n.s.—not significant.

**Table 7:** Differences in Pi*Z and Pi*S allele frequencies among wheezing children, control group, COPD patients from the Central–Eastern European AAT Network, and Lithuanian non-disease specific epidemiological studies (NDSES).

Alleles	Alleles Frequencies for 1000 Alleles (95% CI)			
	Wheezing Group	Control Group	COPD Patients	Lithuanian NDSES
Pi*Z	44.8 (95% CI: 32.1–57.5)	20.27 (95% CI: 11.53–29.01)	46.1 (95% CI: 31.1–60.9)	13.6 (95% CI: 10.7–17.4)
Pi*S	10.3 (95% CI: 4.0–16.6)	20.27 (95% CI: 11.53–29.01)	15.8 (95% CI: 6.92–24.6)	15.6 (95% CI: 12.5–19.6)

CI—95% confidence interval; NDSES—Lithuanian non-disease-specific epidemiological studies, COPD—chronic obstructive pulmonary disease.

## Data Availability

All data generated or analyzed during this study were included in this article. In addition, the datasets used or analyzed during the current study are available on request.
